# Significance of Gram's Stain in Rapid Intrapartum Screening for Maternal Carriership of Group B Streptococcus

**DOI:** 10.1155/S1064744995000421

**Published:** 1995

**Authors:** Albert H. Adriaanse, Harry L. Muytjens, Louis A. A. Kollée, Jan G. Nijhuis, Jacomina A. A. Hoogkamp-Korstanje

**Affiliations:** 1 Department of Obstetrics ad Gynecology University Hospital Nijmegen Nijmegen The Netherlands; 2 Department of Medical Microbiology University Hospital Nijmegen Nijmegen The Netherlands; 3 Department of Pediatrics University Hospital Nijmegen Nijmegen The Netherlands; 4 Department of Obstetrics and Gynecology University Hospital of Amsterdam Academic Medical Centre, Meibergdreef 9 Amsterdam 1105 AZ The Netherlands

## Abstract

*Objective:* Group B streptococcus (GBS, *Streptococcus agalactiae*) is an important cause of neonatal sepsis. Prevention is possible by intrapartum screening for maternal GBS carriership and antimicrobial treatment of colonized women with risk factors during labor. The conflicting results of diagnostic performance are reported both for the newly developed rapid GBS antigen tests and Gram's stain.

*Methods:* The value of Gram's stain in GBS screening was investigated prospectively in 1,020 women. Intrapartum Gram's stains of the cervix from these women and of the introitus from 510 of them were compared with cultures of the cervix, introitus, and anorectum in a semiquantitative way.

*Results:* The sensitivities of the cervical and introital Gram's stains were 25% and 31%, respectively, and the specificities 99% and 98%, respectively. Higher sensitivities (52% and 44%, respectively) were found in heavily colonized parturients. No significant influence of rupture of the membranes was detected. There was a poor correlation between the number of gram-positive cocci in the Gram's stain and the growth density.

*Conclusions:* We do not recommend the routine use of the Gram's stain for intrapartum GBS detection because of both the limited sensitivity and positive predictive value.


Infectious Diseases in Obstetrics and Gynecology 3:110–115 (1995)

© 1995 Wiley-Liss, Inc.

### Significance of Gram's Stain in Rapid Intrapartum Screening for Maternal Carriership of Group B Streptococcus

**Albert H. Adriaanse, Harry L. Muytjens, Louis A.A. Kollée, Jan G. Nijhuis, and Jacomina A.A. Hoogkamp-Korstanje**

*Departments of Obstetrics and Gynecology (A.H.A., J.G.N.), Medical Microbiology (H.L.M., J.A.A.H.-K.), and Pediatrics (L.A.A.K.), University Hospital Nijmegen, Nijmegen, The Netherlands*

### Abstract

***Objective:*** **Group B streptococcus (GBS, *Streptococcus agalactiae*) is an important cause of neonatal sepsis. Prevention is possible by intrapartum screening for maternal GBS carriership and antimicrobial treatment of colonized women with risk factors during labor. The conflicting results of diagnostic performance are reported both for the newly developed rapid GBS antigen tests and Gram's stain.**

***Methods:*** **The value of Gram's stain in GBS screening was investigated prospectively in 1,020 women. Intrapartum Gram's stains of the cervix from these women and of the introitus from 510 of them were compared with cultures of the cervix, introitus, and anorectum in a semiquantitative way.**

***Results:*** **The sensitivities of the cervical and introital Gram's stains were 25% and 31%, respectively, and the specificities 99% and 98%, respectively. Higher sensitivities (52% and 44%, respectively) were found in heavily colonized parturients. No significant influence of rupture of the membranes was detected. There was a poor correlation between the number of gram-positive cocci in the Gram's stain and the growth density.**

***Conclusions:*** **We do not recommend the routine use of the Gram's stain for intrapartum GBS detection because of both the limited sensitivity and positive predictive value.** © 1995 Wiley-Liss, Inc.

Key words

*Streptococcus agalactiae*, gram-positive cocci, sensitivity and specificity

Early onset group B streptococcus (GBS) infections are an important cause of morbidity and mortality in neonates.[1,2] The important perinatal risk factors are heavy maternal GBS colonization, preterm delivery and related low birth weight, preterm rupture of the membranes, prolonged rupture of the membranes (>12–24 h before delivery), intrapartum fever, maternal GBS urinary tract infection, and low levels of maternal serum anti-GBS antibodies.[1–5]

The maternal GBS carrier state plays a crucial role in detecting deliveries at risk for GBS-related disease. Since antepartum cultures are of limited value in that the carriership may be intermittent or temporary,[6] intrapartum cultures in selective broth are the "gold standard."[1,2] However, the culture results are not available until 12–48 h later, which is often too late for decision making. Therefore, rapid GBS screening tests have been developed, including latex agglutination and enzyme immunoassay tests, which are based on direct identification of the group-specific polysaccharide antigen of GBS.[7–26] These tests may be rapid and specific, but their low sensitivity and relatively high costs

Address correspondence/reprint requests to Dr. Albert H. Adriaanse, Department of Obstetrics and Gynecology, University Hospital of Amsterdam, Academic Medical Centre, Meibergdreef 9, 1105 AZ Amsterdam, The Netherlands.

Clinical Study

Received January 19, 1995
Accepted August 9, 1995

are major disadvantages. By contrast, the Gram's stain, being rapid, simple, inexpensive, and readily available, could be used as a screening tool, provided that the sensitivity and specificity are acceptable.

The literature is equivocal regarding the merits of the Gram's stain in intrapartum maternal GBS screening.[16,25,27–31] We investigated the value of the Gram's stain of specimens from different anatomical sites of the maternal genital tract for GBS detection in a prospective study, with special attention to risk factors for GBS disease.

### MATERIALS AND METHODS

### Study Population

The trial, which was part of another study, was conducted from February 1, 1991, until December 1, 1992, in the two hospitals with obstetrical services in our city. The exclusion criteria were known GBS carriership, use of antibiotics during the 4 weeks before admission, planned cesarean delivery, antepartum fetal death, suspected congenital abnormalities, and immature labor. The gestational age, time of sampling, rupture of the membranes, and delivery were recorded. The protocol was approved by both institutional ethical committees and written informed consent was obtained from every parturient enrolled in the trial.

### Collection of Specimens

Four cotton-tipped standard swabs were taken in a fixed rank order from 1,020 women. First, an anorectal culture swab was taken, after which a second culture swab was used to sample the posterior half of the introitus. With a sterile speculum, a third and fourth swab were rotated 360° in the cervical os to obtain specimens for preparing a Gram's stain and for culturing, respectively. After 510 parturients were enrolled, an extra (second-in-rank order) swab was introduced to sample the introitus for another Gram's stain.

The swabs for preparing the Gram's stains were kept in sterile tubes after sampling. The culture swabs, already moistened with Todd-Hewitt broth (Oxoid, Basingstoke, England) before sampling, were put in 5 ml of Todd-Hewitt broth immediately in order to provide a favorable medium. All swabs were stored at 4°C to prevent bacterial overgrowth and processed within 24 h in the same laboratory.

### Laboratory Methods

#### *Gram's Stain*

The Gram's stains were processed and the results recorded by one experienced technician before the culture results were available. The result was reported as positive if gram-positive cocci were identified in clumps, pairs, chains, or individually. For a semiquantitative assessment, the average number of gram-positive cocci per field (1,000×; at least 10–15 nonadjacent fields) was recorded.

#### *Culture Methods*

The culture swabs were streaked on 5% defibrinated sheep blood agar and put into a selective enrichment broth (Todd-Hewitt broth, Oxoid CM 189, Basingstoke, England) with nalidixic acid (0.0015%) and gentamicin (0.0008%) for aerobic bacteria including GBS. The swabs were also cultured for anaerobic bacteria, lactobacilli, and yeast on Casman agar with 10 units/ml of bacitracin and 5% defibrinated rabbit blood (anaerobically, 48 h at 36°C) and Sabouraud agar (72 h at 30°C), respectively. The plates for GBS were incubated at 36°C for 48 h, and the enrichment broth was subcultured after overnight incubation at 36°C onto blood agar and incubated at 36°C for another 24 h. The cultures were judged representative if they reflected normal flora including gram-positive rods representing lactobacilli. The growth of GBS was judged 0–4, corresponding with growth only after enrichment (0) or with growth density in the streak areas as light (1), intermediate (2), or heavy colonization (3 and 4). GBS were identified by colony morphology, a positive CAMP-reaction, an absence of a zone of inhibition around a bacitracin disc (0.04 units) on Casman blood agar (18–24 h at 36°C), and a positive latex agglutination for group B (Streptex; Wellcome Diagnostics, Dartford, England).

### Statistical Methods

The diagnostic performance of the cervical and introital Gram's stain was analyzed with calculation of the sensitivity, specificity, positive predictive value, and negative predictive value. The degree of correlation between cervical Gram's stain and culture was assessed using the Spearman correlation coefficient.

### RESULTS

A total of 981 women was evaluable, 475 of whom had both a cervical and an introital Gram's stain.

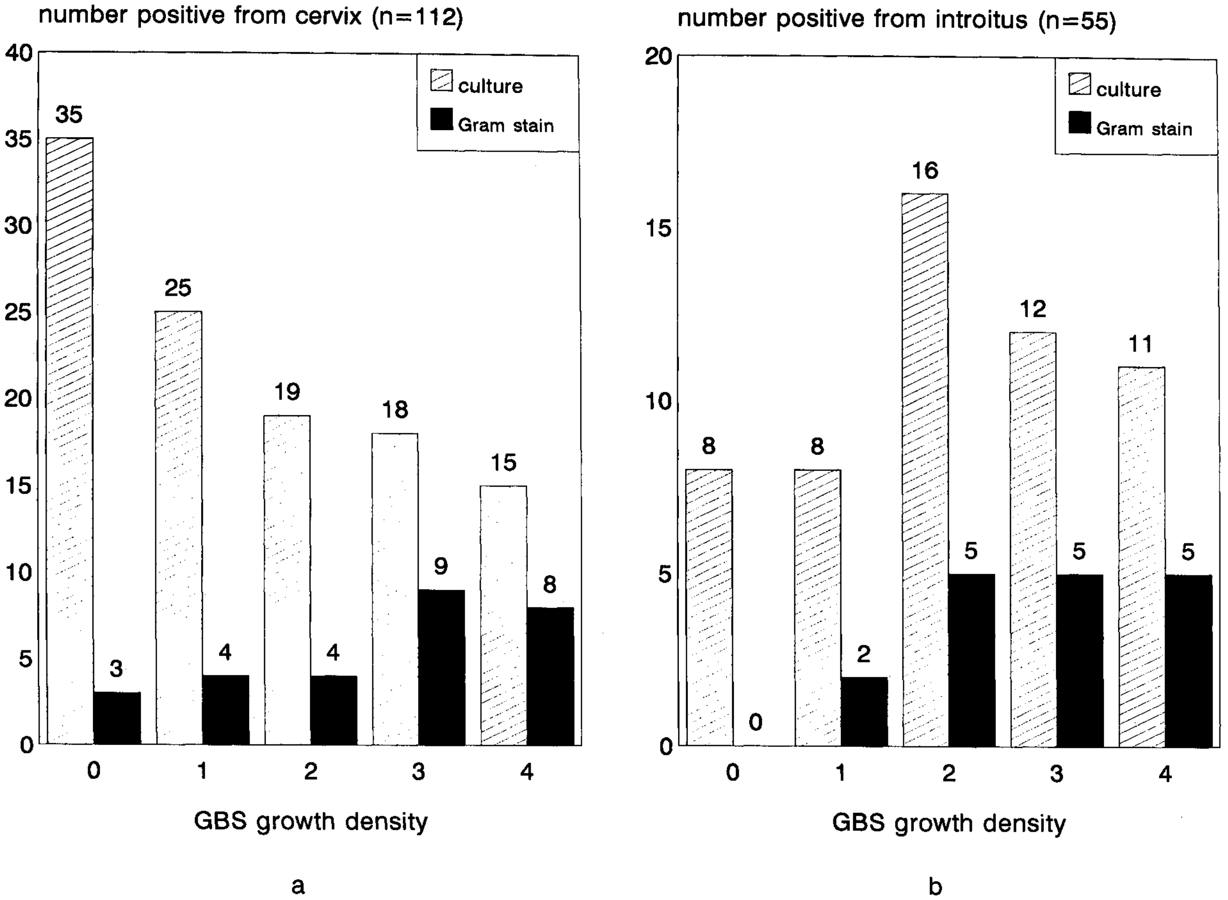

**Fig. 1.** Gram's stain results by GBS growth density in cervical (a) and introital (b) carriers.

The median interval between sampling and parturition was 3.5 h. Overall, GBS colonization was established by culture in 190 (19.4%) women, 108 (11.0%) being heavily colonized. Anorectal, introital, and cervical colonization was found in 169 (17.2%), 139 (14.2%), and 112 (11.4%), respectively; heavy colonization was found in 82 (8.4%), 53 (5.4%), and 33 (3.4%), respectively.

The cervical Gram's stain detected 28 (25%) of 112 cervical carriers (Fig. 1a) and proved false positive in 7 women. The introital Gram's stain detected 17 (31%) of the 55 introital carriers [Fig. 1b; the Gram's stain of the cervix of these women was positive in 8 (15%) of the 52 cervical carriers] and proved false positive in 7 women. As can be seen in the figure, the higher growth densities corresponded with better screening results.

The sensitivity, specificity, and positive and negative predictive values of the cervical and introital Gram's stain are given in Table 1. The specificity of the Gram's stain was quite good. However, the overall sensitivity was only 25.0% for the cervix and 30.9% for the introitus. Higher sensitivities were found for both sites in heavily colonized women.

The amniotic membranes were ruptured before sampling in 72.0% of the parturients. The diagnostic performance of the cervical and introital Gram's stain was not affected by rupture of the membranes (Table 1).

The correlation between the quantitation of gram-positive cocci in the cervical Gram's stain and the growth density was poor, as reflected by a Spearman correlation coefficient of 0.44 (Table 2).

### DISCUSSION

The quick and accurate intrapartum detection of GBS carriers is the Achilles' heel of current preventive strategies with antibiotics, treating colonized parturients if risk factors are present. Rapid

**TABLE 1. Diagnostic performance of Gram's stain compared with selective culture for identification of group B streptococcus**

| | Site (no.) | Sensitivity (%) | Specificity (%) | Predictive value Positive (%) | Predictive value Negative (%) |
|---|---|---|---|---|---|
| Overall | Cervix (981) | 25.0 | 99.2 | 80.0 | 91.1 |
| | Introitus (475) | 30.9 | 98.3 | 70.8 | 91.6 |
| Heavy colonization[a] | Cervix (981) | 51.5 | 98.1 | 48.6 | 98.3 |
| | Introitus (475) | 43.5 | 96.9 | 41.7 | 97.1 |
| Before ROM[b] | Cervix (211) | 11.1 | 100.0 | 100.0 | 92.3 |
| | Introitus (85) | 30.0 | 98.7 | 75.0 | 91.4 |
| After ROM[b] | Cervix (500) | 30.5 | 99.1 | 81.8 | 91.4 |
| | Introitus (238) | 28.6 | 98.1 | 66.7 | 91.2 |

[a]Growth density 3 or 4 on blood agar.
[b]Rupture of membranes (limited to cases in which moment is known with certainty).

**TABLE 2. Correlation between cervical Gram's stain organism quantitation (1,000×; average number of gram-positive cocci of at least 10–15 nonadjacent fields) and selective culture group B streptococcus (GBS) growth density[a]**

| | Selective culture GBS growth density | | | | | | |
|---|---|---|---|---|---|---|---|
| Gram's stain | Negative | 0 | 1 | 2 | 3 | 4 | Total |
| Negative | 862 | 32 | 21 | 15 | 9 | 7 | 946 |
| 0–1 | 6 | 1 | 4 | 1 | 5 | 3 | 20 |
| 2–5 | 1 | 1 | 0 | 3 | 3 | 2 | 10 |
| 6–10 | 0 | 1 | 0 | 0 | 0 | 1 | 2 |
| 11–25 | 0 | 0 | 0 | 0 | 1 | 0 | 1 |
| > 25 | 0 | 0 | 0 | 0 | 0 | 2 | 2 |
| Total | 869 | 35 | 25 | 19 | 18 | 15 | 981 |

[a]Spearman correlation coefficient 0.44.

GBS antigen tests, using samples of the vaginal introitus in 769 women comparable to the population in the present study, yielded an overall sensitivity of 11% and of 25% in heavily colonized women only.[26] Considering this poor sensitivity, a reevaluation of the Gram's stain seems justified.

The diagnostic performance of the Gram's stain was not expected to be very high because the level of detection is $>10^4$–$10^5$ bacteria/ml of sample fluid compared with $\geqslant 10^2$ bacteria/ml of sample fluid for a culture in selective broth, the "gold standard." We found sensitivities of 25% and 31% for the Gram's stain of the cervix and introitus, respectively. The specificities were 99 and 98%, respectively, remarkably higher than in other studies reported thus far. Although these values do not differ significantly, the introitus seems to be the most suitable site for genital sampling because the introital colonization rate was higher than the cervical colonization rate. Moreover, in another study, we found the GBS vertical transmission rate to be highest in women with introital colonization.[32] In addition, introital sampling is more convenient for the patient.

Holls et al.[27] advocated the use of the cervical Gram's stain as an appropriate test to assist in evaluation of GBS colonization in an at-risk population and reported a sensitivity and specificity of 93 and 69%, respectively. Feld and Harrigan,[28] using a vaginal Gram's stain in a selected high-risk population, found a sensitivity of 100% and a specificity of 66.7%, compared with vaginal culture results. The study of Sandy et al.,[29] however, does not support the idea that the cervicovaginal Gram's stain could be a useful screening tool because the sensitivity and specificity were 38 and 61%, respectively, in a preterm population presenting with preterm rupture of the membranes or preterm labor. In 1990, Carey et al.[30] concluded from their results that most gram-positive cocci seen in the

Gram's stain are probably anaerobes or micrococci and that the vaginal Gram's stain at delivery is neither sensitive (34%) nor specific (72%) enough to be valuable as a tool in the diagnosis of maternal GBS carriage. In a comparative study with an enzyme-linked immunosorbent assay, Towers et al.[16] reported a sensitivity of 45% and a specificity of 63% for the cervicovaginal Gram's stain of a high-risk population. In 1993, Hagay et al.[25] made a similar comparison and found a sensitivity of 20% and a specificity of 89% for the endocervical Gram's stain. Therefore, they did not recommend it as a screening test. Some of the differences in the sensitivity and specificity of the Gram's stain reported in the literature may be explained by the level of contamination with gram-positive vaginal flora. Cervical specimens could well be less contaminated than introital specimens, the rectum serving as a GBS reservoir.

Heavy genital GBS colonization is associated with a higher rate of vertical transmission and is a well known risk factor for early onset neonatal sepsis. Towers et al.,[16] studying 20 GBS carriers, found that the sensitivity of the Gram's stain did not change as the colony count increased, in contrast to the increased sensitivity of a rapid enzyme-linked immunosorbent assay test. Our results in 112 cervical and 55 introital carriers, however, indicate that the sensitivity of the Gram's stain is also higher in heavily colonized women. These data are supported by those of "The Vaginal Infections and Prematurity Study Group," reported by Carey et al.,[30] who found a sensitivity of 40% for heavy colonization and an overall sensitivity of 28%.

For future studies, Holls et al.[27] suggested that the Gram's stain organism quantitation be compared with the culture-demonstrated level of colonization. Making this comparison, we found a poor correlation (Table 2). Carey et al.[30] found no correlation between the number of gram-positive cocci or even their presence and the isolation of GBS in laboring women, but a strong correlation with the isolation of *Gardnerella vaginalis* and the impression of bacterial vaginosis.

Prematurity is another major risk factor for early onset neonatal sepsis. Gestational age is not expected to influence the diagnostic performance of the Gram's stain. Carey et al.[30] found an association between the isolation of GBS and gram-positive cocci on the Gram's stain in patients with preterm labor, but the correlation was not great enough to be of clinical value.

Rupture of the amniotic membranes may cause a "wash-out" effect, reducing the genital bacterial colonization.[33] The duration of this effect is unknown. In studying the influence of the membrane status on the performance of the Gram's stain, we could not detect significant differences before or after rupture. Carey et al.[30] did find an association between the presence of gram-positive cocci and the isolation of GBS in the presence of ruptured membranes, but the predictive value was again too small to be of clinical value.

We conclude that, although the diagnostic performance of the Gram's stain seems comparable to that of present rapid GBS antigen tests for intrapartum GBS detection both in sensitivity and specificity, its routine use cannot be recommended because of its limited sensitivity and positive predictive value.

### ACKNOWLEDGMENTS

The authors are indebted to Anton F.J. de Haan, MSc, Department of Medical Statistics, and Hannie G.R. Roelofs-Willemse, Department of Medical Microbiology, for their excellent technical assistance. This study was supported by grant 28-2011 from the Dutch Praeventiefonds.

### REFERENCES

1. Edwards MS, Baker CJ: *Streptococcus agalactiae* (Group B streptococcus). In Mandell GL, Douglas RG Jr, Bennet JE (eds): Principles and Practice of Infectious Diseases. New York: Churchill Livingstone, pp 1554–1563, 1990.
2. Sweet RL, Gibbs RS: Group B streptococci. In Sweet RL, Gibbs RS (eds): Infectious Diseases of the Female Genital Tract. Baltimore: Williams & Wilkins, pp 22–37, 1990.
3. Gerards LJ, Cats BP, Hoogkamp-Korstanje JAA: Early neonatal group B streptococcal disease: Degree of colonisation as an important determinant. J Infect 11:119–124, 1985.
4. Faxelius G, Bremme K, Christensen KK, Christensen P, Ringertz S: Neonatal septicemia due to group B streptococci—Perinatal risk factors and outcome of subsequent pregnancies. J Perinat Med 16:423–430, 1988.
5. Baker CJ, Kasper DL: Correlation of maternal antibody deficiency with susceptibility to neonatal group B streptococcal infection. N Engl J Med 294:753–756, 1976.
6. Hoogkamp-Korstanje JAA, Gerards LJ, Cats BP: Maternal carriage and neonatal acquisition of group B streptococci. J Infect Dis 145:800–803, 1982.

7. Morales WJ, Lim D: Reduction of group B streptococcal maternal and neonatal infections in preterm pregnancies with premature rupture of membranes through a rapid identification test. Am J Obstet Gynecol 157:13–16, 1987.
8. Isada NB, Grossman JH: A rapid screening test for the diagnosis of endocervical group B streptococci in pregnancy: Microbiologic results and clinical outcome. Obstet Gynecol 70:139–141, 1987.
9. Wald ER, Dashefsky B, Green M, Harger J, Parise M, Korey C, Byers C: Rapid detection of group B streptococci directly from vaginal swabs. J Clin Microbiol 25: 573–574, 1987.
10. Tuppurainen N, Hallman M: Prevention of neonatal group B streptococcal disease: Intrapartum detection and chemoprophylaxis of heavily colonized parturients. Obstet Gynecol 73:583–587, 1989.
11. Lotz-Nolan L, Amato T, Iltis J, Wallen W, Packer B: Evaluation of a rapid latex agglutination test for detection of group B streptococci in vaginal specimens. Eur J Clin Microbiol Infect Dis 8:289–293, 1989.
12. Hoppe JE, Lindenau C, Höfler W: Rapid detection of group B streptococci in vaginal swabs of parturients by latex particle agglutination. Zentralbl Bakteriol Mikrobiol Hyg [A] 270:379–384, 1989.
13. Brady K, Duff P, Schilhab JC, Herd M: Reliability of a rapid latex fixation test for detecting group B streptococci in the genital tract of parturients at term. Obstet Gynecol 73:678–681, 1989.
14. Stiller RJ, Blair E, Clark P, Tinghitella T: Rapid detection of vaginal colonization with group B streptococci by means of latex agglutination. Am J Obstet Gynecol 160: 566–568, 1989.
15. Kontnick CM, Edberg SC: Direct detection of group B streptococci from vaginal specimens compared with quantitative culture. J Clin Microbiol 28:336–339, 1990.
16. Towers CV, Garite TJ, Friedman WW, Pircon RA, Nageotte MP: Comparison of a rapid enzyme-linked immunosorbent assay test and the Gram stain for detection of group B streptococcus in high-risk antepartum patients. Am J Obstet Gynecol 163:965–967, 1990.
17. Skoll MA, Mercer BM, Baselski V, Gray JP, Ryan G, Sibai BM: Evaluation of two rapid group B streptococcal antigen tests in labor and delivery patients. Obstet Gynecol 77:322–326, 1991.
18. Gentry YM, Hillier SL, Eschenbach DA: Evaluation of a rapid enzyme immunoassay test for detection of group B streptococcus. Obstet Gynecol 78:397–401, 1991.
19. Greenspoon JS, Fishman A, Wilcox JG, Greenspoon RL, Lewis W: Comparison of culture for group B streptococcus versus enzyme immunoassay and latex agglutination rapid tests: Results in 250 patients during labor. Obstet Gynecol 77:97–100, 1991.
20. Granato PA, Petosa MT: Evaluation of a rapid screening test for detecting group B streptococci in pregnant women. J Clin Microbiol 29:1536–1538, 1991.
21. Clark P, Armer T, Duff P, Davidson K: Assessment of a rapid latex agglutination test for group B streptococcal colonization of the genital tract. Obstet Gynecol 79:358–363, 1992.
22. Green M, Dashefsky B, Wald ER, Laifer S, Harger J, Guthrie R: Comparison of two antigen assays for rapid intrapartum detection of vaginal group B streptococcal colonization. J Clin Microbiol 31:78–82, 1993.
23. Armer T, Clark P, Duff P, Saravanos K: Rapid intrapartum detection of group B streptococcal colonization with an enzyme immunoassay. Am J Obstet Gynecol 168:39–43, 1993.
24. Wüst J, Hebisch G, Peters K: Evaluation of two enzyme immunoassays for rapid detection of group B streptococci in pregnant women. Eur J Clin Microbiol Infect Dis 12:124–127, 1993.
25. Hagay ZJ, Miskin A, Goldchmit R, Federman A, Matzkel A, Mogilner BM: Evaluation of two rapid tests for detection of maternal endocervical group B streptococcus: Enzyme-linked immunosorbent assay and Gram stain. Obstet Gynecol 82:84–87, 1993.
26. Adriaanse AH, Muytjens HL, Kollée LAA, Nijhuis JG, Eskes TKAB: Sensitivity of intrapartum group B streptococcal screening and in vitro comparison of four rapid antigen tests. Eur J Obstet Gynecol Reprod Biol 56:21–26, 1994.
27. Holls WM, Thomas J, Troyer V: Cervical Gram stain for rapid detection of colonization with β-streptococcus. Obstet Gynecol 69:354–357, 1987.
28. Feld SM, Harrigan JT: Vaginal Gram stain as an immediate detector of group B streptococci in selected obstetric patients. Am J Obstet Gynecol 156:446–448, 1987.
29. Sandy EA II, Blumenfeld ML, Iams JD: Gram stain in the rapid determination of maternal colonization with group B beta-streptococcus. Obstet Gynecol 71:796–798, 1988.
30. Carey JC, Klebanoff MA, Regan JA: Evaluation of the Gram stain as a screening tool for maternal carriage of group B beta-hemolytic streptococci. Obstet Gynecol 76: 693–697, 1990.
31. Yancey MK, Armer T, Clark P, Duff P: Assessment of rapid identification tests for genital carriage of group B streptococci. Obstet Gynecol 80:1038–1047, 1992.
32. Adriaanse AH, Kollée LAA, Muytjens HL, Nijhuis JG, de Haan AFJ, Eskes TKAB: Randomized study of vaginal chlorhexidine disinfection during labor to prevent vertical transmission of group B streptococci. Eur J Obstet Gynecol Reprod Biol 61:135–141, 1995.
33. Henderson CE, Egre H, Turk R, Aning V, Szilagyi G, Divon MY: Amniorrhexis lowers the incidence of positive cultures for group B streptococci. Am J Obstet Gynecol 168:624–625, 1993.

## References

[PDF_00517] Adriaanse A. H., Kollée L. A., Muytjens H. L., Nijhuis J. G., de Haan A. F., Eskes T. K. (1995). Randomized study of vaginal chlorhexidine disinfection during labor to prevent vertical transmission of group B streptococci.. Eur J Obstet Gynecol Reprod Biol.

[PDF_00495] Adriaanse A. H., Muytjens H. L., Kollée L. A., Nijhuis J. G., Eskes T. K. (1994). Sensitivity of intrapartum group B streptococcal screening and in vitro comparison of four rapid antigen tests.. Eur J Obstet Gynecol Reprod Biol.

[PDF_00482] Armer T., Clark P., Duff P., Saravanos K. (1993). Rapid intrapartum detection of group B streptococcal colonization with an enzyme immunoassay.. Am J Obstet Gynecol.

[PDF_00410] Baker C. J., Kasper D. L. (1976). Correlation of maternal antibody deficiency with susceptibility to neonatal group B streptococcal infection.. N Engl J Med.

[PDF_00443] Brady K., Duff P., Schilhab J. C., Herd M. (1989). Reliability of a rapid latex fixation test for detecting group B streptococci in the genital tract of parturients at term.. Obstet Gynecol.

[PDF_00510] Carey J. C., Klebanoff M. A., Regan J. A. (1990). Evaluation of the Gram stain as a screening tool for maternal carriage of group B beta-hemolytic streptococci. The Vaginal Infections and Prematurity Study Group.. Obstet Gynecol.

[PDF_00474] Clark P., Armer T., Duff P., Davidson K. (1992). Assessment of a rapid latex agglutination test for group B streptococcal colonization of the genital tract.. Obstet Gynecol.

[PDF_00406] Faxelius G., Bremme K., Kvist-Christensen K., Christensen P., Ringertz S. (1988). Neonatal septicemia due to group B streptococci--perinatal risk factors and outcome of subsequent pregnancies.. J Perinat Med.

[PDF_00503] Feld S. M., Harrigan J. T. (1987). Vaginal gram stain as an immediate detector of group B streptococci in selected obstetric patients.. Am J Obstet Gynecol.

[PDF_00463] Gentry Y. M., Hillier S. L., Eschenbach D. A. (1991). Evaluation of a rapid enzyme immunoassay test for detection of group B Streptococcus.. Obstet Gynecol.

[PDF_00402] Gerards L. J., Cats B. P., Hoogkamp-Korstanje J. A. (1985). Early neonatal group B streptococcal disease: degree of colonisation as an important determinant.. J Infect.

[PDF_00471] Granato P. A., Petosa M. T. (1991). Evaluation of a rapid screening test for detecting group B streptococci in pregnant women.. J Clin Microbiol.

[PDF_00478] Green M., Dashefsky B., Wald E. R., Laifer S., Harger J., Guthrie R. (1993). Comparison of two antigen assays for rapid intrapartum detection of vaginal group B streptococcal colonization.. J Clin Microbiol.

[PDF_00466] Greenspoon J. S., Fishman A., Wilcox J. G., Greenspoon R. L., Lewis W. (1991). Comparison of culture for group B streptococcus versus enzyme immunoassay and latex agglutination rapid tests: results in 250 patients during labor.. Obstet Gynecol.

[PDF_00490] Hagay Z. J., Miskin A., Goldchmit R., Federman A., Matzkel A., Mogilner B. M. (1993). Evaluation of two rapid tests for detection of maternal endocervical group B streptococcus: enzyme-linked immunosorbent assay and gram stain.. Obstet Gynecol.

[PDF_00522] Henderson C. E., Egre H., Turk R., Aning V., Szilagyi G., Divon M. Y. (1993). Amniorrhexis lowers the incidence of positive cultures for group B streptococci.. Am J Obstet Gynecol.

[PDF_00500] Holls W. M., Thomas J., Troyer V. (1987). Cervical Gram stain for rapid detection of colonization with beta-streptococcus.. Obstet Gynecol.

[PDF_00413] Hoogkamp-Korstanje J. A., Gerards L. J., Cats B. P. (1982). Maternal carriage and neonatal acquisition of group B streptococci.. J Infect Dis.

[PDF_00439] Hoppe J. E., Lindenau C., Höfler W. (1989). Rapid detection of group B streptococci in vaginal swabs of parturients by latex particle agglutination.. Zentralbl Bakteriol Mikrobiol Hyg A.

[PDF_00423] Isada N. B., Grossman J. H. (1987). A rapid screening test for the diagnosis of endocervical group B streptococci in pregnancy: microbiologic results and clinical outcome.. Obstet Gynecol.

[PDF_00451] Kontnick C. M., Edberg S. C. (1990). Direct detection of group B streptococci from vaginal specimens compared with quantitative culture.. J Clin Microbiol.

[PDF_00435] Lotz-Nolan L., Amato T., Iltis J., Wallen W., Packer B. (1989). Evaluation of a rapid latex agglutination test for detection of group B streptococci in vaginal specimens.. Eur J Clin Microbiol Infect Dis.

[PDF_00416] Morales W. J., Lim D. (1987). Reduction of group B streptococcal maternal and neonatal infections in preterm pregnancies with premature rupture of membranes through a rapid identification test.. Am J Obstet Gynecol.

[PDF_00506] Sandy E. A., Blumenfeld M. L., Iams J. D. (1988). Gram stain in the rapid determination of maternal colonization with group B beta-streptococcus.. Obstet Gynecol.

[PDF_00459] Skoll M. A., Mercer B. M., Baselski V., Gray J. P., Ryan G., Sibai B. M. (1991). Evaluation of two rapid group B streptococcal antigen tests in labor and delivery patients.. Obstet Gynecol.

[PDF_00447] Stiller R. J., Blair E., Clark P., Tinghitella T. (1989). Rapid detection of vaginal colonization with group B streptococci by means of latex agglutination.. Am J Obstet Gynecol.

[PDF_00454] Towers C. V., Garite T. J., Friedman W. W., Pircon R. A., Nageotte M. P. (1990). Comparison of a rapid enzyme-linked immunosorbent assay test and the Gram stain for detection of group B streptococcus in high-risk antepartum patients.. Am J Obstet Gynecol.

[PDF_00431] Tuppurainen N., Hallman M. (1989). Prevention of neonatal group B streptococcal disease: intrapartum detection and chemoprophylaxis of heavily colonized parturients.. Obstet Gynecol.

[PDF_00427] Wald E. R., Dashefsky B., Green M., Harger J., Parise M., Korey C., Byers C. (1987). Rapid detection of group B streptococci directly from vaginal swabs.. J Clin Microbiol.

[PDF_00486] Wüst J., Hebisch G., Peters K. (1993). Evaluation of two enzyme immunoassays for rapid detection of group B streptococci in pregnant women.. Eur J Clin Microbiol Infect Dis.

[PDF_00514] Yancey M. K., Armer T., Clark P., Duff P. (1992). Assessment of rapid identification tests for genital carriage of group B streptococci.. Obstet Gynecol.

